# Characteristic features of the insertions of the distal tibiofibular ligaments on three-dimensional computed tomography- cadaveric study -

**DOI:** 10.1186/s40634-020-0220-6

**Published:** 2020-01-23

**Authors:** Sho Kikuchi, Goro Tajima, Atsushi Sugawara, Jun Yan, Moritaka Maruyama, Shinya Oikawa, Takaaki Saigo, Ryunosuke Oikawa, Minoru Doita

**Affiliations:** 1 Department of Orthopedic Surgery, Iwate Medical University, Uchimaru, 19-1, Morioka, Iwate Japan; 2 Department of Anatomy, Iwate Medical University, Uchimaru,19-1, Morioka, Japan

**Keywords:** Syndesmotic joint, Anterior tibiofibular ligament, Posterior tibiofibular ligament, Three-dimensional images, Osseous landmarks

## Abstract

**Purpose:**

The purpose of this study was to clarify the insertion sites of the anterior inferior tibiofibular ligament (AITFL) and posterior inferior tibiofibular ligament (PITFL) and related osseous landmarks on three-dimensional computed tomography images.

**Methods:**

Twenty-nine non-paired, formalin-fixed human cadaveric ankles were evaluated. The tibial and fibular insertion sites of the AITFL and PITFL were identified. The morphology and location of the insertion sites and their positional relationships with osseous structures were analyzed on three-dimensional computed tomography images.

**Results:**

The AITFL had a trapezoidal shape, with fibers that ran obliquely lateral from a wider insertion at the lateral distal tibia to the medial distal fibula. The PITFL had a similar shape to the AITFL; however, it ran more horizontally, with fibers running in the same direction. In the tibia, the anterior capsular ridge and the Chaput’s and Volkmann’s tubercles were useful osseous landmarks for the insertion sites. In the fibula, the centers of the insertion sites of the AITFL and PITFL were located on the edges of the distal anterior and posterior fibula, which were useful osseous landmarks. The mean distances between the center points of the tibial and fibular insertion sites of the AITFL and PITFL were 10.1 ± 2.4 mm and 11.7 ± 2.6 mm, respectively.

**Conclusions:**

The relationships between the characteristic features of the distal tibia and fibula and the insertions of the AITFL and PITFL were consistent. The present findings improve the understanding of the anatomy of the insertions of the distal tibiofibular syndesmotic joint.

## Background

A syndesmotic joint is a fibrous joint in which a strong membrane or ligaments link two adjacent bones without articular cartilage. The distal tibiofibular syndesmotic joint is formed by the distal tibia, distal fibula, and four ligaments: the anterior inferior tibiofibular ligament (AITFL), posterior inferior tibiofibular ligament (PITFL), inferior transverse ligament, and interosseous ligament [6]. This fibrous joint plays a very important role in ankle joint stability and motion. The functions of the distal tibiofibular syndesmotic ligaments are to stabilize the ankle mortise, provide integrity between the distal tibia and the fibula, and enable complex movement of the talus [8].

Distal tibiofibular syndesmotic joint injuries are often caused by sports injuries, including ankle fractures. In fractures of the ankle, syndesmotic joint injury reportedly occurs in all Weber C fractures and in about 50% of Weber B fractures that occur due to high-energy trauma [5]. Instability of the syndesmotic ligaments leads to abnormal movement of the talus within the mortise and abnormal loading of the articular surfaces. Widening of the ankle mortise can lead to severe instability and decrease the contact area of the tibiotalar articulation, which can result in chronic ankle pain and progressive degeneration of the articular surfaces of the ankle [5, 9].

Several new surgical techniques for both the acute and chronic phases have recently been reported, with superior results compared with traditional surgical procedures [2, 4, 7, 8, 12, 13, 16]. Anatomical suture-button fixation in the optimal direction (from the posterior cortex of the fibula to the anterolateral edge of the tibia) provides adequate stabilization compared with other procedures [13]. AITFL anatomical augmentation using suture tape may also be a useful option in the acute phase [12]. Additionally, anatomical reconstruction of the AITFL for chronic instability has shown good clinical results in both objective and subjective evaluations [4, 8, 16]. Anatomical reconstruction of the PITFL has also been a topic of research because of its importance in ankle stability [3, 7].

Several anatomical studies of the ankle syndesmotic ligaments have been reported [1, 3, 6, 14]. However, little has been mentioned about the actual positions of these ligamentous insertions and the relationships between these positions and osseous landmarks; therefore, they are still controversial. To perform either anatomical augmentation or reconstruction of the AITFL and PITFL, it is necessary to precisely define the anatomical positions of their insertion sites and the relationships among the structures making up the distal tibiofibular joint.

The aim of the present study was to describe the insertion sites of the AITFL and PITFL and the related osseous landmarks on three-dimensional (3-D) computed tomography (CT) images. We hypothesized that there are consistent, identifiable, characteristic features of the AITFL and PITFL insertion sites.

## Materials and methods

### Specimen preparation

Twenty-nine ankles from human cadavers were used. All specimens had a normal appearance and no history of trauma, surgery, severe degenerative changes, and/or other ankle abnormalities. The mean age at the time of death was 75.2 ± 9.8 years (range, 50–89 years). All cadavers had been fixed in 10% formalin and preserved in 50% alcohol for 6 months. These cadavers were donated to Iwate Medical University for education and research purposes, and informed consent for donation was obtained from each patient and their family prior to death. This cadaveric study was approved by our Institutional Review Board (approval number: H27–99).

Dissection began with removal of the skin and subcutaneous soft tissue on the lateral side of the ankle. The tendons, muscles, and fascia were carefully removed to expose the ligamentous structures. A detailed dissection was performed to accurately identify the ligamentous structures of the ankle syndesmotic joint, including the AITFL and PITFL, and the relevant bony structures. After identification, these ligaments were bisected in half, and the insertion sites of the AITFL and PITFL (defined as the areas where the ligament fibers arose from the tibia and fibula) were outlined using a fine 1.0-mm-diameter drill, with care taken to prevent destruction of peripheral structures in the subcutaneous soft tissue. We excluded the interosseous ligament from this study because few surgical techniques for anatomical augmentation and reconstruction of the interosseous ligament have been reported.

### Three-dimensional measurements and visualization

**Fig. 1:**
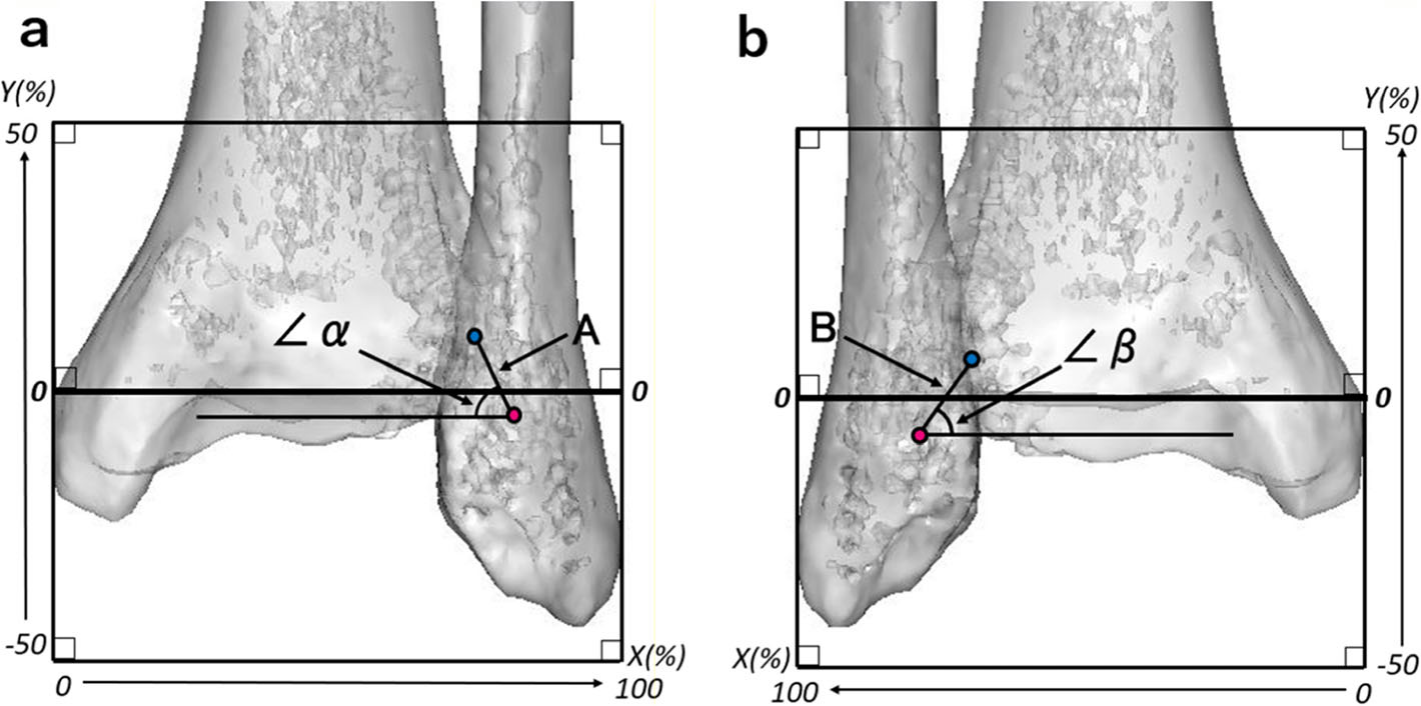
Measurements of the angles and lengths of the ligaments and original coordinate planes with squares. Distances between the center points of the tibial and fibular insertion sites of the (**a**) anterior inferior tibiofibular ligament (AITFL) and (**b**) posterior inferior tibiofibular ligament (PITFL). The angles were formed between a horizontal line and the line between the center points of the tibial and fibular insertions of the AITFL (α) and PITFL (β). The maximum medial–lateral distance between the medial tibial cortical line and most of the lateral fibular cortical line in the true anterior view of the three-dimensional images was used as a standard (100%), and coordinate grids fitting the medial and lateral condyles on the three-dimensional images were created. The x-axis was defined as the middle of the coordinate grid (the horizontal line of the tibial plafond). The y-axis was defined as the medial perpendicular line on the grids. The origin of the coordinate axes was the point of intersection between the y-axis and the horizontal line of the tibial plafond

The ankles were scanned using a 16-row multislice CT scanner (ECLOS®; Hitachi Medical Corporation, Tokyo, Japan). Axial sections with 0.675-mm thickness were acquired and saved as Digital Imaging and Communications in Medicine (DICOM) data. All data were uploaded to dedicated software (Mimics® version 19.0 and MedCAD® module; Materialise N.V., Leuven, Belgium) and reformatted into 3-D images. The tibial and fibular insertion sites of the AITFL and PITFL and related osseous structures were analyzed on the 3-D images. The abovementioned software was used to automatically define the centers of the insertions as the center of their surfaces [10, 11]. We automatically measured the lengths and widths of these insertion sites and the distances between the center points of the tibial and fibular insertion sites. We also measured the angles formed by a horizontal line running along the tibial plafond and the line between the center points of the tibial and fibular insertions of the AITFL and PITFL (Fig. 1). The coordinates of the centers of the tibial and fibular insertion sites of the AITFL and PITFL were mapped on coordinate grids in the true anterior and posterior views on the 3-D images (Fig. 1). The maximum medial–lateral distance between the medial tibial cortical line and most of the lateral fibular cortical line in the true anterior view of the 3-D images was used as a standard (100%). These 3-D measurements were based on the methods described by Saigo et al. [10] and Takahashi et al. [11].

### Statistical analysis

The distribution of each variable was checked for normality using the Kolmogorov–Smirnov test. Statistical data were calculated using commercial software (SPSS version 20.0®; IBM Corp., Armonk, NY, USA).

## Results

### Macroscopic findings

**Fig. 2:**
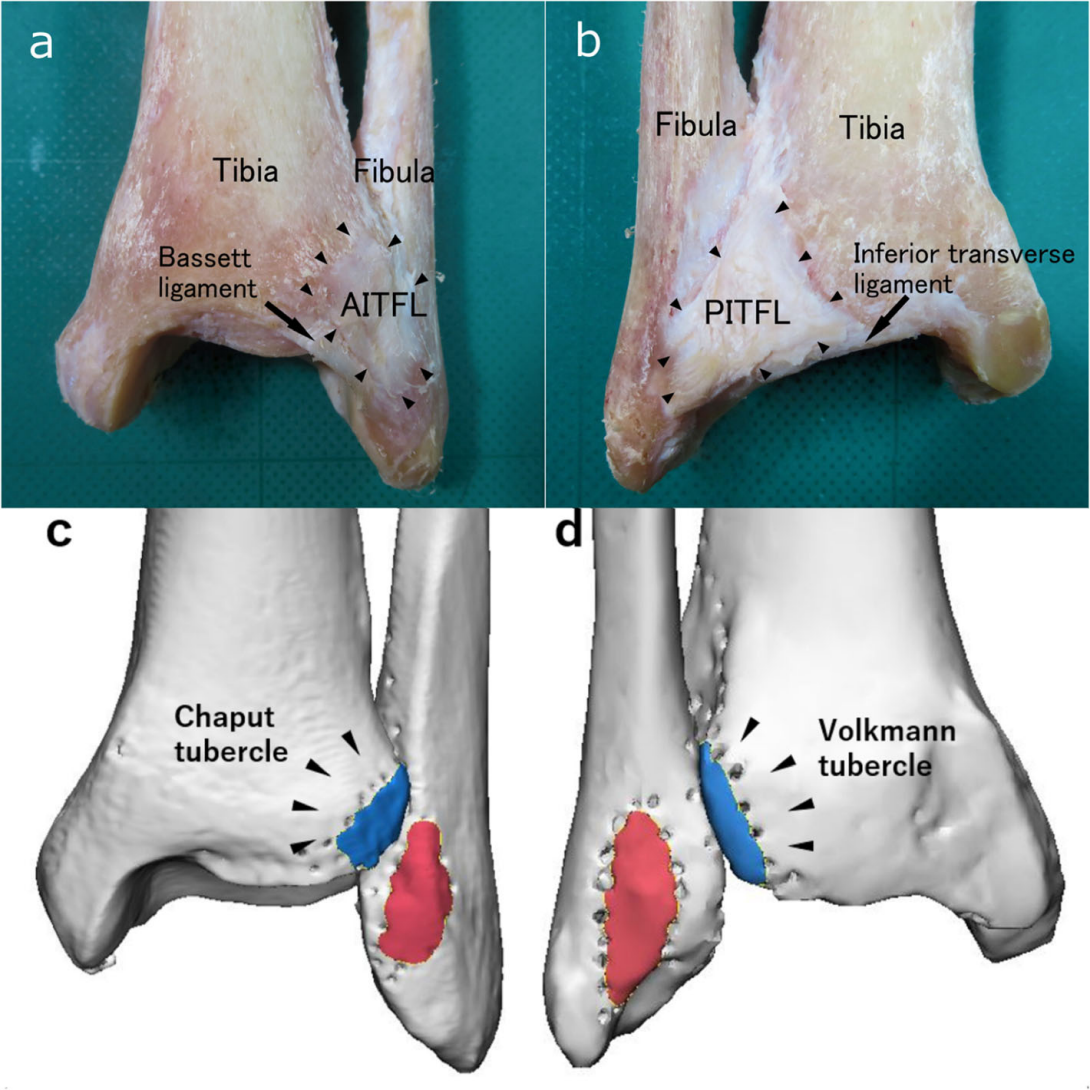
Specimen photographs and three-dimensional computed tomography images of the ankle. **a** Anterior and (**b**) posterior photographs. **c** Anterior and (**d**) posterior views of the three-dimensional computed tomography images. AITFL = anterior inferior tibiofibular ligament; PITFL = posterior inferior tibiofibular ligament

Both the AITFL and PITFL were clearly identified in all specimens. The AITFL had a trapezoidal shape because its tibial insertion was wider than the fibular attachment, and its fibers ran obliquely from medial to lateral to the distal fibula (Fig. 2a). The distal fascicle of the AITFL, Bassett’s ligament [15], ran obliquely inferior and inserted on the anteromedial aspect of the fibula and parallel to the ATIFL. The insertions of the longer fascicles were closer to the origin of the AITFL. The PITFL had a similar shape to the AITFL; however, it ran more horizontally, and its fibers ran obliquely from medial at the distal tibia to lateral at the distal fibula (Fig. 2b). The deep fibers of the PITFL, the inferior transverse ligament, originated from the posteromedial aspect of the tibial plafond and inserted on the posteromedial aspect of the fibula.

### Three-dimensional analysis of the syndesmotic joint

**Fig. 3:**
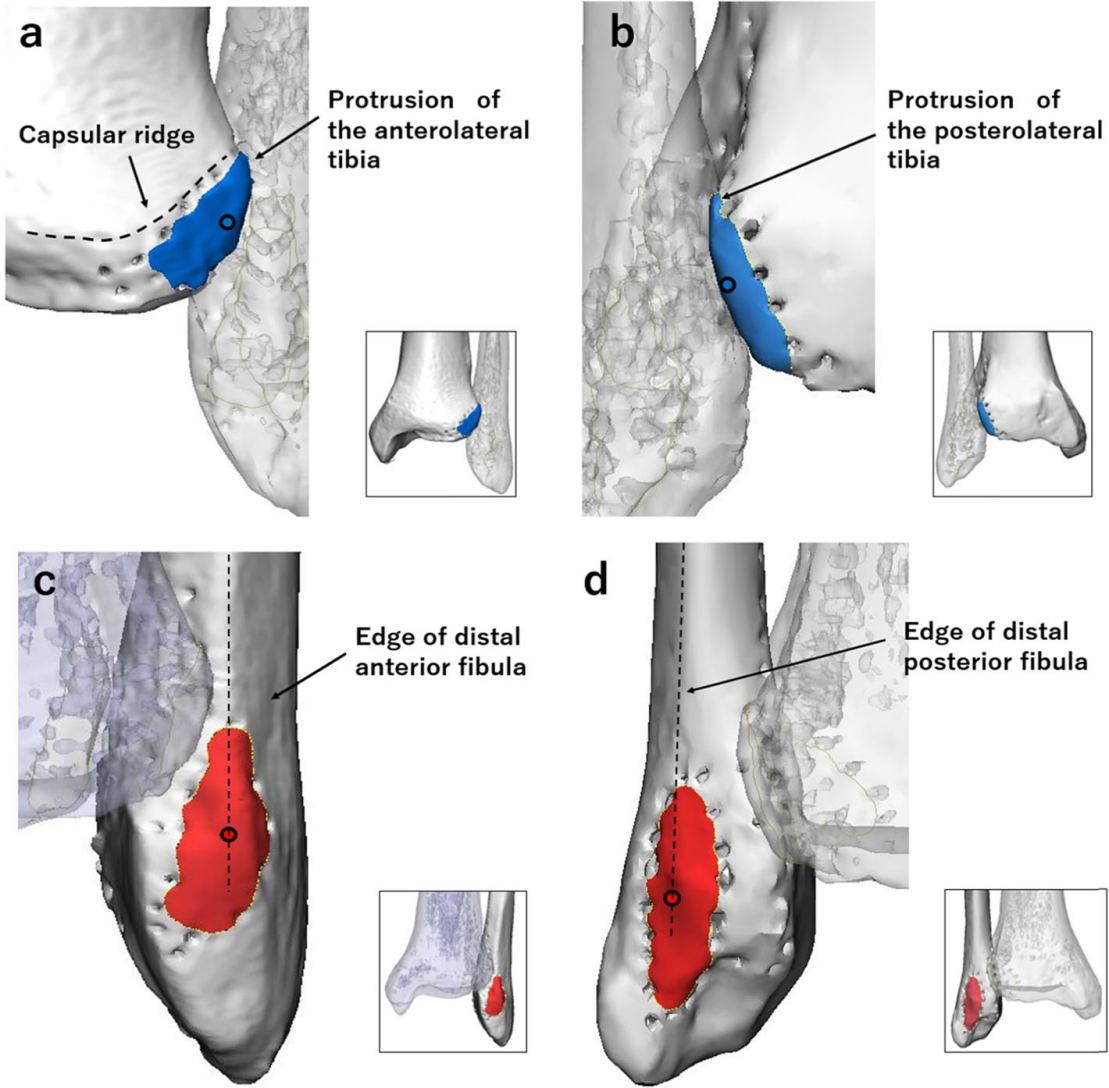
Osseous landmarks on three-dimensional images of the distal tibiofibular syndesmosis. **a** Tibial insertion site of the anterior inferior tibiofibular ligament (AITFL). **b** Tibial insertion site of the posterior inferior tibiofibular ligament (PITFL). **c** Fibular insertion site of the AITFL. **d** Fibular insertion site of the PITFL

Three-dimensional images of the distal tibiofibular joint were analyzed to identify the insertion sites, and their centers, of the AITFL and PITFL. On the tibial side, the insertion site of the AITFL was inferior and lateral to the anterior capsular ridge and distal to the anterolateral tibial tubercle (Chaput’s tubercle) (Figs. 2c, 3a). The insertion site of the PITFL was distal to the posterolateral tibial tubercle (Volkmann’s tubercle) (Figs. 2d, 3b). On the fibular side, the center points of the AITFL and PITFL insertions were on the edges of the distal anterior and posterior fibula, respectively (Fig. 3c, d). The insertion sites were both elliptical in shape. The center points of the tibial insertion sites of the AITFL and PITFL were more proximal than the center points of the fibular insertion sites.

The mean lengths and widths of the tibial and fibular insertions of the AITFL and PITFL are shown in Table 1. The mean distances between the center points of the tibial and fibular insertion sites of the AITFL and PITFL were 10.1 ± 2.4 mm and 11.7 ± 2.6 mm, respectively. The mean angles formed by the tibial plafond and the lines between the center points of the tibial and fibular insertion sites of the AITFL and PITFL were 67.1° ± 10.0° and 50.5° ± 13.1°, respectively (Table 2).

**Table 1:** Lengths and widths of the insertion sites of the anterior inferior tibiofibular ligament and posterior inferior tibiofibular ligament

	Anterior inferior tibiofibular ligament		Posterior inferior tibiofibular ligament	
	Tibial side	Fibular side	Tibial side	Fibular side
Length (mm)	16.4 ± 2.3 (10.7–21.5)	15.4 ± 2.4 (12.1–20.6)	17.3 ± 2.5 (11.2–21.6)	17.1 ± 3.1 (12.9–24.0)
Width (mm)	7.5 ± 1.6 (4.1–9.6)	7.6 ± 2.0 (4.9–12.0)	7.4 ± 1.4 (5.1–9.8)	7.5 ± 1.7 (5.0–12.3)

Data are presented as mean ± standard deviation (range)

**Table 2:** Mean distances and angles between the center points of the tibial and fibular insertion sites of the AITFL and PITFL

Distance (mm)		Angle	
AITFL	PITFL	AITFL	PITFL
10.1 ± 2.4 (5.2–16.0)	11.7 ± 2.6 (6.3–17.0)	67.1 ± 10.0° (51–85)	50.5 ± 13.1° (36–70)

Data are presented as mean ± standard deviation (range)

**Fig. 4:**
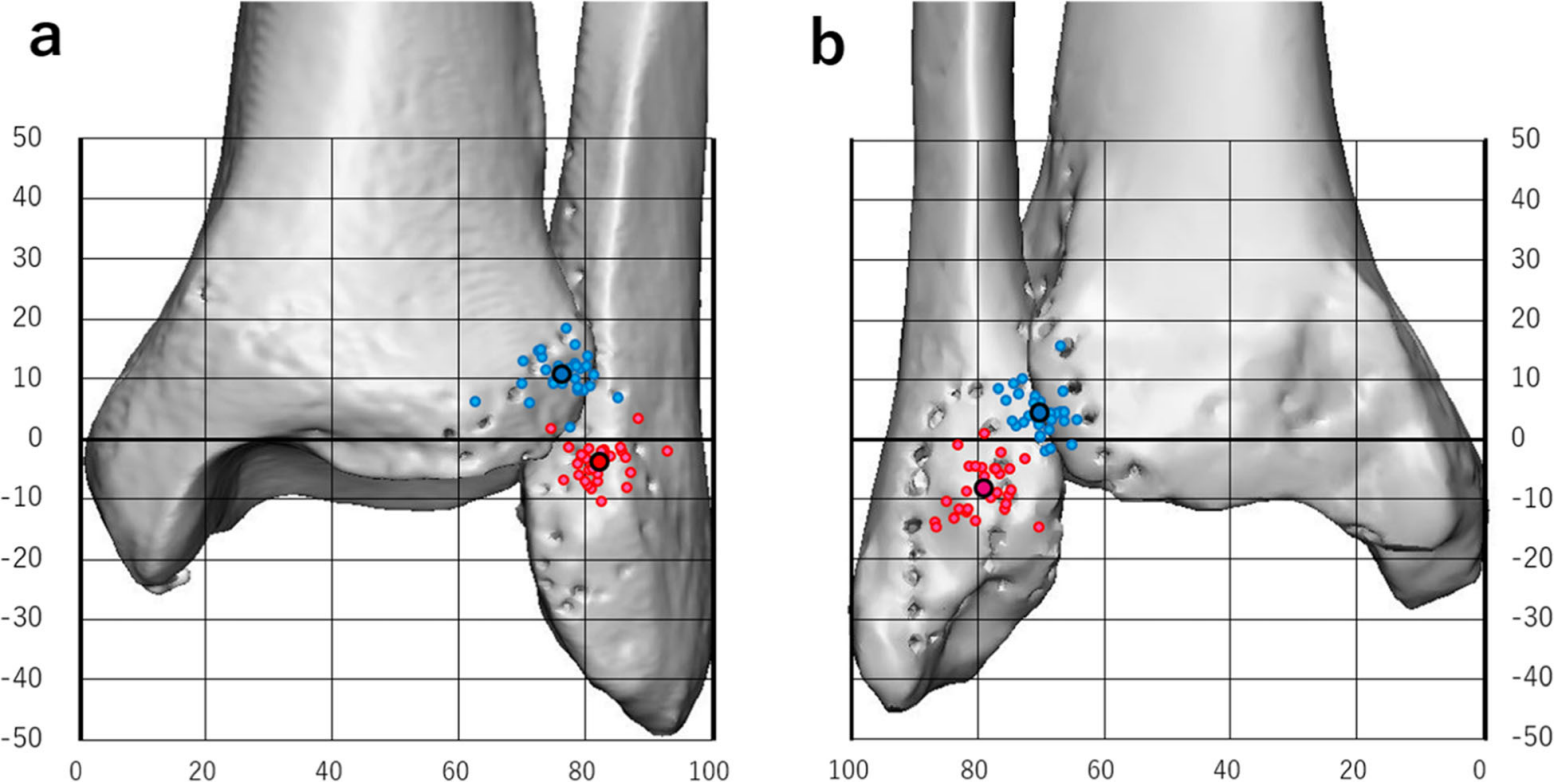
Coordinate maps of the insertions of the ligaments. Coordinates for the centers of the tibial (blue circles) and fibular (red circles) insertion sites of the (**a**) anterior inferior tibiofibular ligament and (**b**) posterior inferior tibiofibular ligament The large blue circle and red circle indicate the mean centers of the insertion sites of these ligaments.

Coordinates were obtained for the centers of the AITFL and PITFL insertion sites (Fig. 4), and their locations on true anterior and posterior two-dimensional images are summarized in Table 3.

**Table 3:** Location coordinates on anterior and posterior views of three-dimensional images

	Anterior inferior tibiofibular ligament		Posterior inferior tibiofibular ligament	
	Tibial side	Fibular side	Tibial side	Fibular side
X (%)	76.4 ± 4.3 (62.6–85.2)	82.3 ± 3.8 (74.6–93.1)	70.1 ± 3.2 (64.3–76.7)	79.0 ± 4.0 (70.2–86.7)
Y (%)	10.6 ± 3.3 (1.8–18.3)	−3.9 ± 3.2 (− 10.6–1.7)	4.4 ± 3.7 (− 2.1–15.5)	−8.2 ± 4.2 (− 14.7–1.0)

Data are presented as mean ± standard deviation (range)

## Discussion

The most important findings of the present study are the clarification of the characteristic features of the distal tibia and fibula and the fact that the locations of the insertion sites of the AITFL and PITFL on 3-D images are consistent. These findings will improve the understanding of the anatomy of the insertion sites of the AITFL and PITFL and assist surgeons in performing anatomical procedures to treat injuries involving these ligaments.

We found several important osseous landmarks for both the fibular and tibial insertion sites of the AITFL and PITFL. On the tibial side, the anterior capsular ridge and the Chaput’s and Volkmann’s tubercles were clear osseous landmarks for the insertion sites of the AITFL and PITFL. On the fibular side, the edges of the distal anterior and posterior fibula, respectively, also provide osseous landmarks for the insertion sites of the AITFL and PITFL. These findings are similar to the findings of a previous study that described the capsular ridge and the tubercles as osseous landmarks, but did not show the detailed relationships between these landmarks [15]. That previous study did not mention the detailed morphology of bone or the relationships between the center points of the insertions of the AITFL and PITFL on 3-D CT images.

We calculated the precise centers of the insertion sites of the AITFL and PITFL and then showed the relationships of these positions using coordinate maps on anterior and posterior views on 3-D images, including angular measurements [10, 11]. No previous studies have described the positions of the centers of the insertion sites of the AITFL and PITFL on 3-D images. Our mapping method used the percentage of the total length, enabling us to minimize the influence of individual differences [10]. Several studies have measured the distances of the ligament insertion sites from the articular cartilage as a reference line using manual calipers or digitizing systems [5, 6, 14]. This reference line is an easily visible intraoperative indicator; however, it may be easily influenced by degenerative changes and is difficult to evaluate on radiographic images. The tibial articular surface line that we adopted can be easily and clearly identified on radiographic images, and so our mapping method may be a useful reference for preoperative planning.

The present study revealed the angles, lengths, and shapes of the AITFL and PITFL using the centers of their insertion sites to mark their course. A previous study using a goniometer reported that the AITFL runs obliquely at a 35° angle in a lateral and distal direction, while the PITFL runs almost horizontally at a 20° angle to the horizontal plane [3]. These previously reported angles are smaller than those measured in the present study. The reason for these differences might be because we measured the angles using the centers of the insertion sites of the ligaments or because of other differences in measurement methods. We also measured the distances between the center points of the ligaments’ tibial and fibular insertion sites. Several studies have reported differing measurements of the ligaments, including the distances between the upper and lower margins and the center margin or the edge-to-edge lengths of the ligaments [1, 3, 6, 14, 15]. No previous study has measured the distance between the center points of the insertion sites of the ligaments, and so our results cannot be compared with previous studies. We described the shapes, and measured the lengths and widths, of the insertion sites as a simple combined bundle. Several studies have reported the lengths and widths of these ligaments divided into several bundles [1, 3, 6, 14, 15]; however, the functions of each bundle are still unclear. We believe that our findings of the ligaments as combined bundles might be more practical for determining graft length and size and for creating optimal bone tunnels in anatomical reconstructive procedures.

Our study had several limitations. First, a relatively small number of specimens was investigated. Second, the cadavers had a high mean age, and so we cannot rule out the influence of degenerative changes. Third, although we used accurate 3-D measurement methods, it is possible that human dissection and subjective decisions introduced error and bias into the subsequent steps. Fourth, we used a drill to mark the outlines of the insertions, so we could not analyze each bundle of the AITFL and PITFL. Fifth, we used formalin-preserved cadavers in which it is occasionally difficult to identify detailed structures and investigate the biomechanics of the distal tibiofibular syndesmotic joint. Further study and consideration will be needed to yield biomechanical and surgical data.

## Conclusions

The present study used 3-D CT images to show the relationships between the insertions of the AITFL and PITFL and osseous landmarks. The clinical relevance of this study is that it may improve the understanding of the anatomy of the insertions of AITFL and PITFL, and thus assist surgeons in performing anatomical reconstruction.

## Data Availability

All data analyzed during this study are included in this published article.

## Abbreviations

3-D: Three-dimensional
AITFL: Anterior inferior tibiofibular ligament
CT: Computed tomography
PITFL: Posterior inferior tibiofibular ligament
